# Bidirectional contraction of a type six secretion system

**DOI:** 10.1038/s41467-019-09603-1

**Published:** 2019-04-05

**Authors:** Piotr Szwedziak, Martin Pilhofer

**Affiliations:** Institute of Molecular Biology & Biophysics, Eidgenössische Technische Hochschule Zürich, CH-8093 Zürich, Switzerland

## Abstract

Contractile injection systems (CISs) mediate cell-cell interactions by a phage tail-like apparatus. Their conserved mechanism relies on the anchoring of the proximal end of a sheath-tube module to a membrane, followed by contraction of the sheath towards the attachment site and ejection of the inner tube. Here we reveal a major variation of the CIS mechanism in the type six secretion system (T6SS) of enteroaggregative *Escherichia coli* (EAEC). We show that both ends of the sheath-tube module are attached to opposite sides of the cell, enabling the structure to contract in two opposite directions. The protein TssA1 mediates the interaction of the distal end with the cell envelope, the termination of tail elongation, and non-canonical contraction towards the distal end. We provide a framework for the molecular processes at the T6SS distal end. Further research will address whether bidirectional contraction allows for bidirectional effector secretion. The unrecognized concept of non-canonical contractions could be relevant to biofilms of the human intestine.

## Abstract (web summary)

Bacteria use contractile injection systems, such as type VI secretion systems (T6SS), to secrete proteins that mediate cell-cell interactions. Here, Szwedziak & Pilhofer show that both ends of a T6SS can attach to opposite sides of the cell, enabling the structure to contract in two opposite directions.

## Introduction

Bacterial contractile injection systems (CISs) mediate diverse cell–cell interactions by employing a phage tail-like apparatus^1^. All CISs are structurally, functionally and evolutionarily related to contractile phages^2,3^. Their conserved mechanistic theme relies on the attachment of one end of a sheath-tube module to a membrane, followed by contraction of the tail sheath toward this proximal attachment site, which in turn ejects the inner tube^4^. CISs function by two different modes of action; either they are released into extracellular space, or they remain in the bacterial cytoplasm. Extracellular CISs, including antibacterial pyocins^5^, metamorphosis-associated contractile structures^6^, and insecticidal antifeeding prophages^7^ are released into the medium upon host cell lysis. The proximal end of their sheath-tube attaches to the surface of the target cell, and contraction leads to puncturing of the target. By contrast, the type six secretion system’s (T6SS’s) proximal end is anchored in the host’s inner membrane (IM) and the T6SS translocates effectors to the target in a cell-cell contact-dependent mechanism, puncturing the cell envelopes of the host and target^8–10^.

A T6SS apparatus comprises several functional modules (Supplementary Fig. 1a). The transenvelope complex TssJLM provides the exit channel during cargo ejection and recruits the tail to the inner membrane^11^. The contractile tail consists of a baseplate and the sheath-tube module^8^. The baseplate is a multi-protein complex that serves as a polymerization platform for the assembly of the sheath-tube and as a connector of the sheath-tube with the transenvelope complex^12^. The Hcp tube is capped with a VgrG/PAAR spike complex and enveloped by the TssB/C sheath^13,14^. Sheath contraction toward the baseplate translocates the effector-loaded tube-spike into the target. TssA has been described as a factor that is required for T6SS assembly and was suggested to be present in the baseplate^15^ or at the distal end of a growing T6SS tail^16^, however, its exact role remains elusive.

During assembly, the proximal end of the T6SS sheath-tube is bound to the baseplate and new subunits are added at the distal end that grows into the cytoplasm^17^. The tail length of classical T6SSs is heterogeneous^8^ and was shown to correlate with cell size in *Vibrio cholerae* spheroplasts^17^ and with the expression level of the inner tube protein Hcp in engineered *V. cholerae* cells^10^. Tape measure and tail terminator proteins that tightly regulate tail length in contractile phages^18^, extracellular CISs^19–21^ and possibly in the divergent T6SS^iv^^2^, are absent from classical T6SS gene clusters. A recent study suggested a role of the T6SS protein TagA in terminating T6SS tail elongation^22^.

The T6SS is one of the most abundant bacterial secretion systems and plays important roles in diverse ecological settings by mediating interactions with eukaryotic and bacterial targets^23^. Shaping bacterial communities in biofilms is one of the key roles of the T6SS. Here we investigated the T6SS-1 in enteroaggregative *Escherichia coli* (EAEC), which has been established as a major T6SS model^11,16,24,25^. By time-lapse imaging, we discovered that the EAEC T6SS-1 not only contracts toward its proximal end but also toward its distal end—a phenomenon that we call non-canonical contraction. To investigate this, we developed a strategy to fluorescently label the inner Hcp tube by means of orthogonal translation. The system was then used for correlative cryo-light/cryo-electron microscopy (CLEM) to image extended T6SSs in situ by electron cryotomography (ECT). The approach revealed bent T6SSs whose proximal and distal ends were associated with opposite sides of the cell. We identified TssA1 as a factor mediating the termination of tail elongation, the anchoring of the T6SS distal end to the cell envelope, and non-canonical contraction toward the distal end.

## Results

### The EAEC T6SS-1 displays non-canonical contraction events

**Fig. 1:**
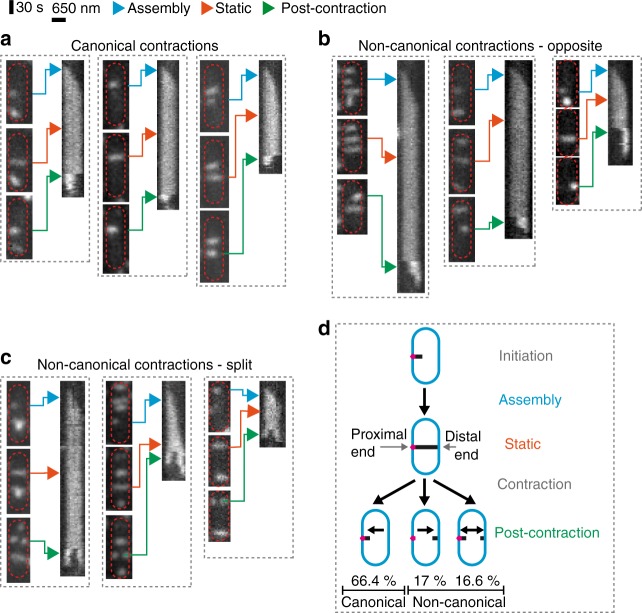
The dynamics of the EAEC T6SS-1 reveal non-canonical contraction events. **a**–**c** EAEC TssB1-sfGFP cells were analyzed by time-lapse fluorescence imaging (1 frame every 3 s). For each example (boxed by grey dashed lines), three snapshots for each cell are shown, representing assembly/static/post-contraction states, respectively. For each example, the three snapshots are correlated with a kymograph of a selected T6SS structure as indicated by arrows. Cell outlines are indicated by red dashed lines. Images were positioned to orient T6SS assembly start sites (proximal ends) on the left. See also Supplementary Movies 1–3. Three classes of contraction events were observed. Canonical contraction (observed previously^8,27^) occurred toward the proximal end (**a**). Non-canonical opposite contraction occurred toward the distal end (**b**), or the T6SS split and contraction occurred simultaneously toward the proximal and distal ends (**c**). **d** Interpretation of the observed EAEC T6SS-1 dynamics. Quantification shows that non-canonical contractions accounted for 33.6% of all measured events (*n* = 593). For statistical analysis see Supplementary Fig. 1b. Blue, cell envelope; magenta, proximal baseplate/transenvelope complex; black, T6SS sheath

In order to characterize the dynamics of the T6SS apparatus, we performed time-lapse imaging of live EAEC cells in which one sheath protein was fluorescently tagged (TssB1-sfGFP^26^). The analysis of fluorescence light microscopy (fLM) data revealed that T6SS structures that were parallel to the imaging plane usually polymerized until they reached the opposite side of the cell and then stopped growing. T6SSs stayed in the extended conformation for an average time of 236 ± 106 s (*n* = 28), before contracting. Interestingly, we observed three distinct patterns of contraction (Fig. 1, Supplementary Fig. 1b, Supplementary Movie 1, 2). The most common pattern represented 66.4% (*n* = 593) and revealed contraction toward the structure’s polymerization start-site at the baseplate (here referred to as the proximal end; Fig. 1a). This pattern had been reported for T6SSs in *V. cholerae*^8^ and EAEC^27^ and represents the canonical model of firing of T6SSs (and CISs in general). Strikingly, 17% of the monitored events showed T6SS sheaths that entirely contracted toward the distal end (Fig. 1b). Another 16.6% of all events accounted for a third pattern of T6SS structures that split into two fragments, which simultaneously contracted toward the proximal end and distal end, respectively (Fig. 1c, d). Canonical contractions (toward proximal end) and non-canonical contractions (toward distal end, or toward both ends) were also observed in EAEC spheroplasts that allowed for the assembly of longer structures (Supplementary Fig. 1c-e, Supplementary Movie 3). Non-canonical contractions in EAEC were likely overlooked in previous studies because imaging experiments were performed at lower temporal resolution (one frame every ~30 s in e.g.^27,28^ vs. 3 s in this study). In accordance with previous reports^8,29^, non-canonical contraction events were absent from *V. cholerae* (Supplementary Fig. 2, Supplementary Movie 4).

### Cell-spanning, bent T6SSs in a non-contractile TssB1 mutant

We reasoned that a major prerequisite for non-canonical contractions would be a physical association of the tail distal end with the cell envelope. To investigate this hypothesis, we set out to image the EAEC T6SS-1 in situ by ECT. Since the imaged cells did not reveal extended T6SSs, we pursued two alternative strategies to capture the EAEC T6SS-1 in the pre-contraction state by (1) imaging a non-contractile sheath mutant and (2) labeling the inner tube to use it as a marker for extended T6SSs.

**Fig. 2:**
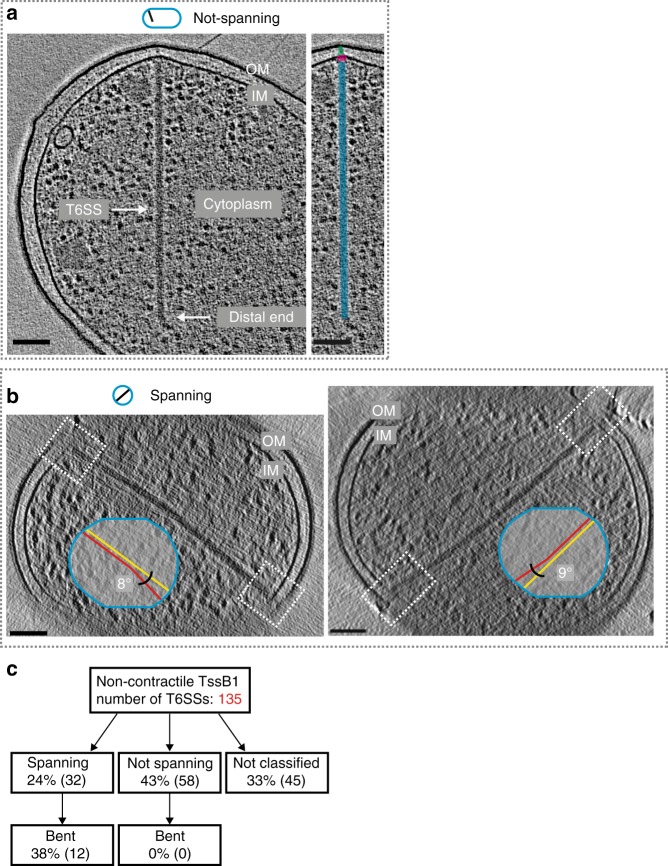
ECT imaging of a non-contractile TssB1 mutant reveals T6SS structures that span the cells and are bent. Shown are tomographic slices (21.6 nm thickness) of three examples of EAEC T6SSs (non-contractile TssB1 mutant) imaged by ECT. Schematics above indicate the viewing angle and approximate orientation of the T6SS in the cell. IM, inner membrane; OM, outer membrane. Scale bars: 100 nm. **a** One class of structures was found with the proximal end attached to the cell envelope and with the distal end ending in the cytoplasm (left panel). The structural modules transenvelope complex (green)/baseplate (magenta)/sheath-tube (cyan) were segmented in the right panel. **b** A second class of T6SSs spanned across the cytoplasm and both ends were associated with opposing membranes. Shown are cross sections (views along the cell’s long axis) of two examples. Spanning T6SSs were frequently bent >5° (illustrated by the schematic insets). White dashed rectangles highlight the areas where the T6SS ends approach the cell envelope. Note that for any given structure, it was impossible to distinguish the proximal and distal ends with high confidence. **c** Diagram quantifying the classification of imaged T6SSs. Note that bending (bending angle defined as >5°) was only observed for spanning structures. T6SSs for which the missing wedge prevented from reliable classification were categorized as “not classified” (see Supplementary Fig. 3)

First, we engineered a non-contractile T6SS by introducing a mutation into the *tssB1* sheath gene, as shown previously for *V. cholerae*^30^. As expected, time-lapse imaging showed static T6SSs and the absence of any contractions (Supplementary Movie 5). Electron cryotomograms of these cells for the first time revealed in situ structures of the EAEC T6SS-1 model system (65% of the imaged cells showed one or more T6SSs, *n* = 174). Since the relatively large diameter of EAEC cells impaired data quality, we performed ECT imaging with a Volta phase plate^31^, resulting in increased contrast. In general, the EAEC T6SS’s structural features were similar to T6SSs of the subtype-1 that were reported previously for *V. cholerae*^8^ and *Myxococcus xanthus*^32^, including the sheath-tube module extending into the cytoplasm, and the presence of baseplate and transenvelope modules (Fig. 2a). Importantly, besides structures whose distal end was found ending in the cytoplasm, ECT imaging also revealed structures that entirely spanned the short-axis of the cell (Fig. 2b). Both sheath-tube ends of such structures were in close proximity to the inner membranes on approximate opposite sides of the cell. A third category of structures could not be assigned to the above classes, since the ECT missing wedge of data obscured one or both ends of the structures (Supplementary Fig. 3). Strikingly, 38% of the spanning T6SSs were clearly bent (structures were considered bent for angles >5°, as defined in Fig. 2b insets), while T6SSs that evidently did not span the entire cell width were always straight (Fig. 2c). We hypothesized that the observation of bending in spanning T6SSs indicates that both ends were physically attached to the cell envelope. Earlier ECT work on *V. cholerae*^8^ and *M. xanthus*^32^ neither reported T6SS bending, nor T6SSs that were spanning the entire cell diameter.

### The T6SS inner tube can be labeled by orthogonal translation

We then sought an alternative strategy to image extended EAEC T6SSs by ECT that would not require the introduction of a mutation into a sheath component. We therefore decided to fluorescently label the inner tube protein (Hcp1) and use it as a reporter for extended T6SSs in a correlative cryo-light/cryo-electron microscopy (CLEM) approach. Because of steric occlusion by the sheath and the small ~2-nm tube lumen^14^, genetic tagging attempts of any Hcp with fluorophores such as GFP have to date been unsuccessful. We therefore resorted to labeling by orthogonal translation, which was previously shown for a bacterial cytoskeletal protein^33,34^. Briefly, the gene of interest is deleted from the chromosome and instead expressed from a plasmid; importantly, an amber stop codon is introduced into the coding sequence. A heterologously expressed orthogonal translation system then incorporates a fluorescent coumarin-derived amino-acid (CouAA; supplied to the culture) at amber codon sites.

**Fig. 3:**
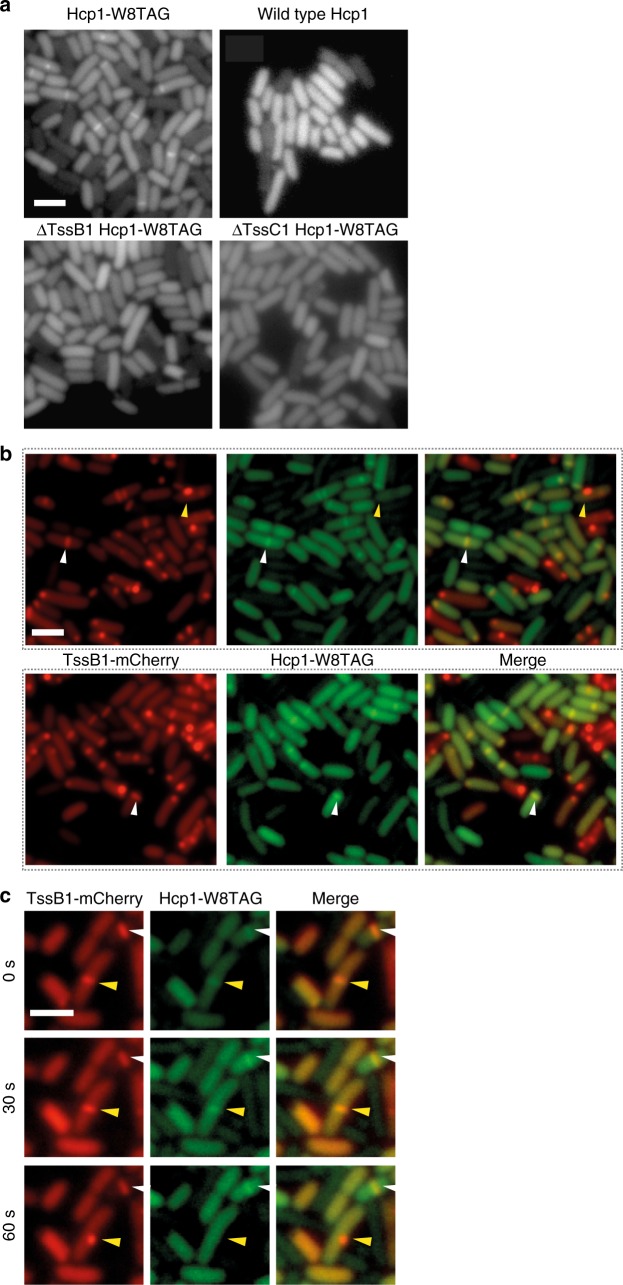
The T6SS inner tube can be labeled by orthogonal translation. **a** Shown is the first successful labeling approach for a T6SS Hcp. An orthogonal translation system was used to synthesize Hcp1-CouAA by the introduction of an amber stop codon (Hcp1-W8TAG) and growing the cells in the presence of fluorescent CouAA. Imaging revealed fluorescent streaks across the cells’ short axes, likely representing T6SSs in the extended conformation (top left panel). The control experiment (wild type Hcp) showed homogeneous signal distribution and therefore no incorporation of CouAA into T6SSs (top right panel). Both sheath components (TssB1/C1) were required for T6SS assembly (bottom panels). All samples were grown in the presence of CouAA. See Supplementary Fig. 4 for a validation of CouAA incorporation into the Hcp1-W8TAG primary structure. **b** Shown is an imaging experiment, combining the orthogonal translation system (middle column) with fluorescently-tagged sheath (TssB1-mCherry, left column). Essentially every Hcp1-CouAA signal co-localized with a sheath signal (white arrowhead), indicating that Hcp1-CouAA was incorporated into T6SSs. Sheath signals that did not co-localize with Hcp1-CouAA signals were likely contracted T6SSs (yellow arrowhead). **c** Time-lapse imaging (time points indicated on left) of the setup in **b** showed that upon contraction of the sheath, Hcp1-CouAA signal was lost, indicating the expulsion of Hcp1 from the cell (yellow arrowhead). The white arrowhead marks a T6SS that did not contract over the time course. Scale bars: 2 μm

We screened eleven different positions for the amber codon in the *hcp1* sequence and found one [TGG(W8)–>TAG(STOP)] that complemented the *hcp1* knockout. Biochemical and mass-spectrometry analyses showed that labeled Hcp1 (Hcp1-CouAA) was secreted into the supernatant of a culture and that the labeling efficiency for full-length Hcp1 was 100% (Supplementary Fig. 4). fLM imaging of labeled cells revealed fluorescent streaks across the cytoplasm (Fig. 3a, top left). These characteristic streaks likely represented extended T6SSs and were absent when the *hcp1* deletion was complemented with wild type *hcp1* rather than with the amber codon-containing version (Fig. 3a, top right). Earlier reports^35,36^ that suggested that the T6SS inner tube only assembles in the presence of sheath protein were supported by the experiment with a sheath knockout background (Fig. 3a, lower panels). To further verify that the fluorescent streaks seen by orthogonal translation indeed corresponded to extended T6SSs, we performed a dual labeling experiment in a fluorescently tagged sheath (TssB1-mCherry) background (Fig. 3b). Essentially every Hcp1 signal co-localized with a signal for labeled sheath. Sheath signals, which did not show a signal for Hcp1, likely represented contracted T6SSs. Time-lapse imaging indicated that Hcp1 was in fact expelled upon contraction (Fig. 3c). The ejected Hcp1 tube probably disassembled, since we never detected any fluorescent streaks in extracellular space. In summary, the shown system presents the first successful labeling attempt of a T6SS Hcp tube and can serve future studies.

### Cell-spanning, bent T6SSs revealed by correlated cryo-fLM/ECT

**Fig. 4:**
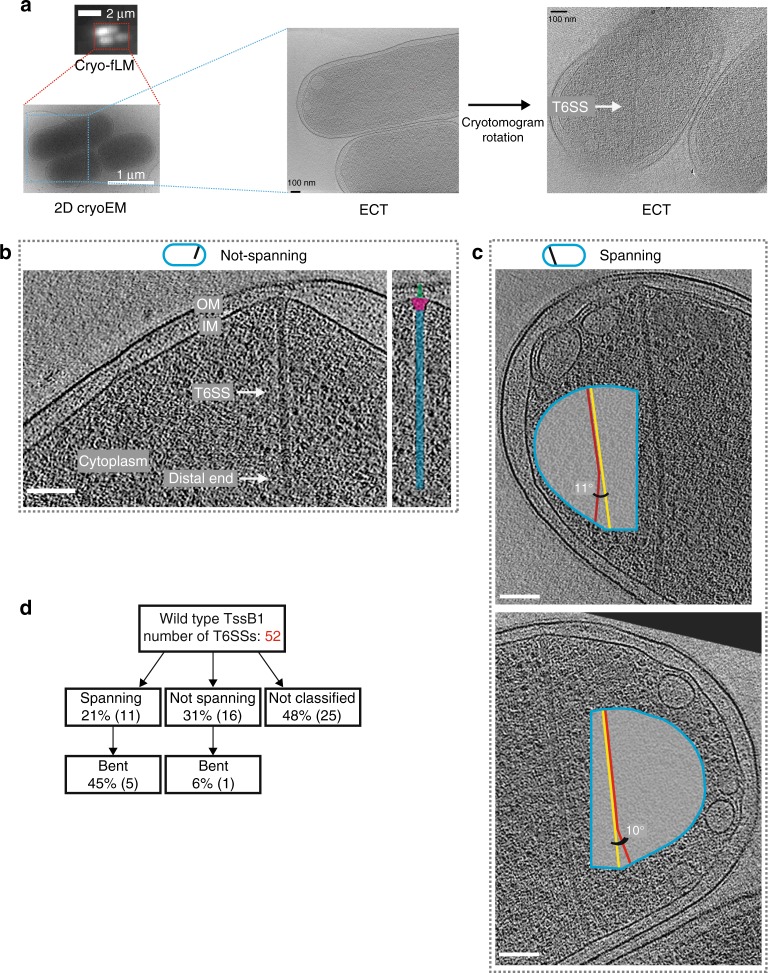
Hcp1-labeling combined with a correlated cryo-fLM/ECT approach reveals T6SSs that span the cell and are bent. **a** Shown is the correlative cryo-light/cryo-electron microscopy (CLEM) workflow. Cells were cultured, labeled with Hcp1-CouAA by orthogonal translation, and plunge-frozen on an EM grid. The frozen grid was imaged in an FEI CorrSight cryo-light microscope with a 40× air objective (example shown, cryo-fLM) to determine coordinates of cells that showed Hcp1-CouAA signal (indicating extended T6SSs). The grid was subsequently transferred to a cryo-transmission EM. Cells were located by 2D imaging at low magnification (2D cryoEM) and imaged by ECT (two different cryotomographic slices are shown). **b**, **c** The orthogonal translation system was used to identify extended T6SSs on a plunge-frozen EM grid. Candidate cells were imaged by ECT. Besides T6SSs whose distal ends were localized in the cytoplasm (**b**), some T6SSs spanned the entire cell, with both ends associated with the IM (**c**). The spanning structures were frequently bent (indicated by insets). Note that the direction of bending enabled the structures to approach both the IMs at an angle closer to 90°. Schematics on top indicate the viewing angle and approximate orientation of the T6SS in the cell. Scale bars: 100 nm. Thickness of tomographic slices: 21.6 nm. **d** Diagram quantifying the classification of imaged T6SSs. Note that bending was primarily observed for spanning structures. T6SSs for which the missing wedge prevented from reliable classification were categorized as “not classified”

Next, we used Hcp labeling as an indicator to select cells with extended T6SSs for ECT imaging. We cultivated EAEC with CouAA-labeled Hcp1 and plunge-froze cells on an electron microscopy (EM) grid. The frozen grid was then imaged by cryo-fLM to identify cells with extended T6SSs. After transferring the grid into a cryo-electron microscope, we were able to narrow down ECT data collection to cells that displayed Hcp1 signal (Fig. 4a). This approach was crucial to reveal 52 extended T6SSs in a wild type sheath background. Interestingly, the cryotomograms revealed again T6SSs whose distal end was found in the cytoplasm (Fig. 4b) and T6SSs that spanned the entire width of the cell with both ends associated with the cell envelope (Fig. 4c). Strikingly, also this imaging approach revealed a high abundance of bending among spanning structures (45%), and not among structures that were only associated to the cell envelope with one end (Fig. 4d). Intriguingly, the bending always occurred in the direction that would result in the angle of the T6SS approaching the membrane being closer to 90° compared to without bending (Fig. 4c).

To summarize, time-lapse fLM imaging and both our ECT imaging strategies suggested that once the polymerizing distal T6SS end reached the opposite side of the cell, the tail stopped growing and its both ends were physically associated with the cell envelope.

### TssA1 mediates elongation termination and non-canonical contraction

We wondered which factor could mediate membrane association of the T6SS distal end. Such a component should be able to bind to the polymerizing sheath-tube end and have an affinity for the IM and/or to a binding partner in the IM. The conserved protein TssA1 is required for T6SS function and could fulfill these requirements. TssA1 was described as an assembly factor that co-localizes with the polymerizing end of the EAEC T6SS-1 and binds to both the transenvelope complex TssJLM^16^ and the membrane-associated TagA protein^22^.

**Fig. 5:**
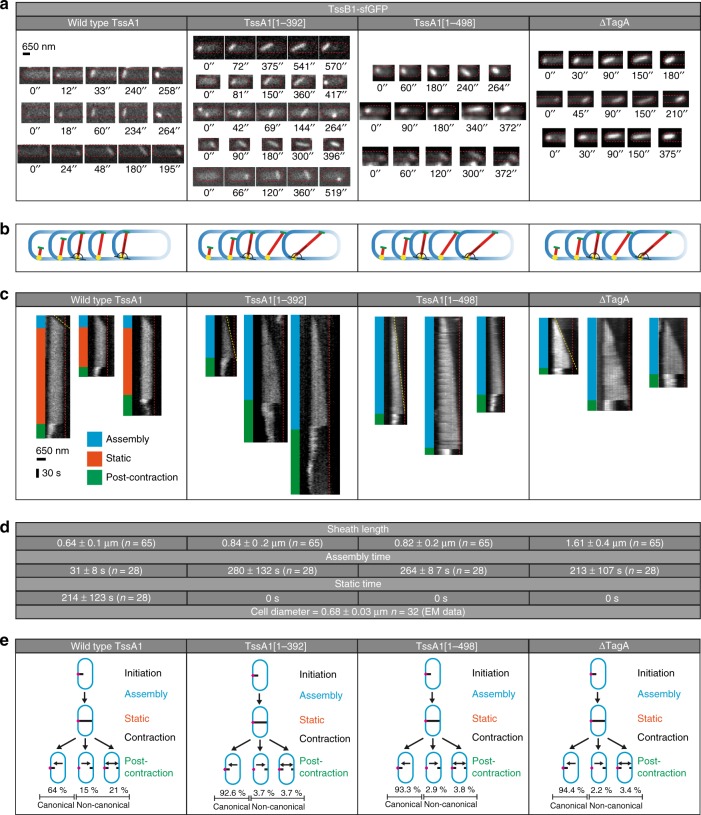
TssA1 mediates tail elongation termination, anchoring the T6SS distal end to the cell envelope, and non-canonical contraction toward the distal end. **a** Shown is a comparison of EAEC TssB1-sfGFP time lapse imaging (time points indicated) in wild type TssA1, TssA1[1–392], TssA1[1–498], and ΔTagA backgrounds, respectively. In the TssA1[1–392], TssA1[1–498], and ΔTagA backgrounds, polymerizing structures that reached the opposite membrane continued to elongate, resulting in sliding of the T6SS distal end against the IM (to accommodate for increasing length) until contraction occurred (last image in each sequence). See Supplementary Movie 6. In contrast, when Δ*tssA1* was complemented with wild type *tssA1*, elongation terminated once the distal end approached the inner membrane and the sliding phenotype was not observed (see also Fig. 1 and Supplementary Movie 2). The proximal end remained static, except when it snapped off the cell envelope resulting in the tail-sheath module floating in the cytoplasm. The distal end in the wild type background became static once the T6SS spanned the entire cell width, whereas the distal end in all the presented mutants was sliding along the IM, leading to a change in the angle of the T6SS with respect to the cell envelope. **b** The shown schematic recapitulates sheath dynamics before contraction that were observed in (**a**). Proximal end, yellow; distal end, green; T6SS sheath, red. **c**, **d** Further analyses of sheath dynamics (kymographs shown in **c** revealed that in the presented mutant backgrounds, structures kept polymerizing (slower than for wild type-TssA1, yellow line slope) until contraction occurred and never entered a static extended phase. Sheaths were on average longer than the cell diameter. In contrast, for wild type TssA1, T6SSs entered a static extended phase upon spanning the entire cell width and the average sheath length was comparable to the average cell diameter. Source data are provided as a Source Data file. **e** The complementation of Δ*tssA* with *tssA1*[1–392] (*n* = 547) or *tssA1*[1–498] (*n* = 209) on a plasmid showed a significantly reduced number of non-canonical contractions, as compared to complementation with wild type *tssA1* on a plasmid (*n* = 143) and wild type *tssA1* in the native locus (Fig. 1d). TssA1 therefore mediates non-canonical contractions. Δ*tagA* (*n* = 90) background also reduced the number of non-canonical contractions

In order to investigate whether TssA1 played a role in the interaction of the T6SS distal end with the cell envelope, we generated a series of TssA1 mutants and supplied them on plasmids in a Δ*tssA1* background (Supplementary Fig. 5). The only two mutants that recovered T6SS assembly (as seen by time lapse imaging of the GFP-tagged sheath) were the deletions of the C-terminal part of the protein, encompassing TssA1 residues 1–392 or 1–498 (below referred to as TssA1[1–392] and TssA1[1–498] mutants, respectively). The TssA1[1–392] mutant lacks the entire dodecameric C-terminal domain^16^ whereas the TssA1[1–498] mutant is deprived of only the EX extension that is specific for the TssA2^B^ family^37^. Strikingly, unlike the TssA1 wild type, once the distal end of an assembling TssA1[1–392]/TssB1-sfGFP or a TssA1[1–498]/TssB1-sfGFP system reached the IM, polymerization was not terminated; instead, the T6SS tail rather continued to elongate. In order to accommodate for the increasing length, the distal end of the T6SS then started to slide against the IM, leading to a more and more shallow angle between the sheath and the cell envelope at both proximal and distal ends, rather than remaining approximately perpendicular (Fig. 5a, b, Supplementary Movie 6). The position of the proximal end remained stable and no sliding was observed here. The TssA1[1–392] and TssA1[1–498] mutants not only differed from the wild type regarding the distal end characteristics. Their sheaths were also longer, assembly time was prolonged, and a static waiting phase in the extended conformation before contraction was absent (Fig. 5c, d). Interestingly, a ΔTagA/TssB1-sfGFP strain displayed a behavior that was similar to the two TssA1 sliding mutants (Fig. 5a–d; also observed in^22^). The combination of wild type TssA1 with the non-contractile TssB1-sfGFP mutant demonstrated that T6SS polymerization was again terminated upon the distal end approaching the opposite cell boundary, resulting in a T6SS average length similar to wild type TssA1/TssB1-sfGFP cells (Supplementary Fig. 6). The inability to contract did therefore not affect length control.

Together, the results support the idea that TssA1 and TagA are both involved in mediating the termination of sheath polymerization once the distal end reaches the IM. Furthermore, attachment of the T6SS distal end to the cell envelope allows for non-canonical contraction events toward the distal end in wild type cells. This is fully consistent with our observation that the fraction of non-canonical contractions was significantly lower in the TssA1[1–392], TssA1[1–498] and ΔTagA mutants (Fig. 5e).

### TssM1 and TagA are required to recruit TssA1 to the cell envelope

**Fig. 6:**
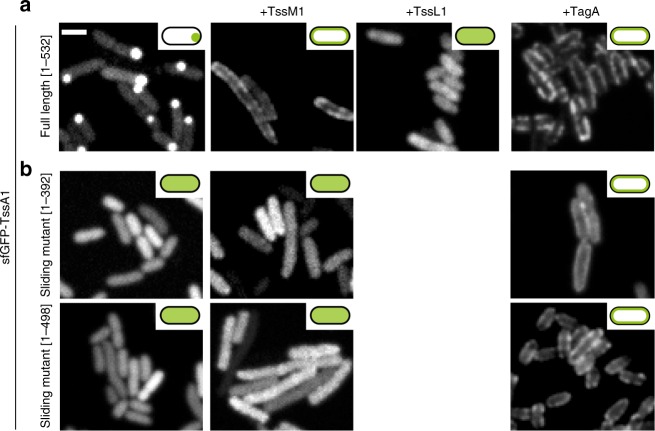
TssM1 and TagA recruit TssA1 to the cell envelope in vivo. Full length (**a**) and truncated (**b**) sfGFP-tagged TssA1 constructs were expressed either alone or together with TssM1/TssL1/TagA. sfGFP-TssA1[1–532] showed strong polar foci that likely represented inclusion bodies, as reported previously^16^. Co-expression of sfGFP-TssA1[1–532] with TssM1 or TagA resulted in a patchy signal at the cell envelope, indicating recruitment to the membrane. The TssA1 C-terminus was essential for the TssM1- but not the TagA-dependent membrane recruitment, as the co-expression of sfGFP-TssA1[1–392] or TssA1[1–498] with TssM1 showed whole cell fluorescence. Both TssA1 sliding mutants could localize to the IM in the presence of TagA but a significant fraction of the labeled proteins was present in the cytoplasm, indicating a lower affinity of the interaction. TssL1 failed to recruit sfGFP-TssA1[1–532] to the membrane. Cellular localization of relevant sfGFP-TssA1 constructs is shown in schematic diagrams. Scale bar: 2 μm

Since TssA1 does not feature any recognizable membrane domains that would allow for direct binding to the cell envelope, it is likely that another factor mediates this interaction. In vitro experiments based on gel filtration and negative stain EM suggested an interaction of TssA1 with the transenvelope complex TssJLM^16^. We also noticed that TssA1 and TssM1 are encoded by overlapping open reading frames in the EAEC *sci-1* gene cluster, suggesting that they are co-regulated. Recent in vivo labeling suggested that TssA1 also interacts with the membrane-associated protein TagA^22^. We therefore probed a possible TssM1- and TagA-dependent recruitment of TssA1 to the membrane in vivo, by monitoring the cellular localization of sfGFP-TssA1 in the absence or presence of TssM1, TssL1 or TagA in heterologous *E. coli* host cells. In the absence of any T6SS component, full length sfGFP-TssA1[1–532] was found to localize in strong polar foci, consistent with the previous observation of inclusion bodies^16^ (Fig. 6a). Importantly, co-expression of full length sfGFP-TssA1[1–532] with TssM1 or TagA resulted in a patchy signal predominantly localized at the cell envelope, while co-expression with TssL1 resulted in a dispersed signal (whole cell fluorescence) (Fig. 6a). The TssM1-mediated recruitment of full length sfGFP-TssA1 to the cell envelope was dependent on TssA1’s C-terminal domain (Fig. 6b), which is fully consistent with the sliding phenotype displayed by TssA1[1–392] and TssA1[1–498] (Fig. 5a–c). In contrast, TagA could still recruit both TssA1 C-terminal truncations to the cell envelope (Fig. 6b).

Results of the TssA1 membrane recruitment assay indicate the complexity of the mechanism where TssM1 and TagA work in conjunction to reliably anchor the TssA1-decorated distal tail-sheath end to the cell envelope.

## Discussion

**Fig. 7:**
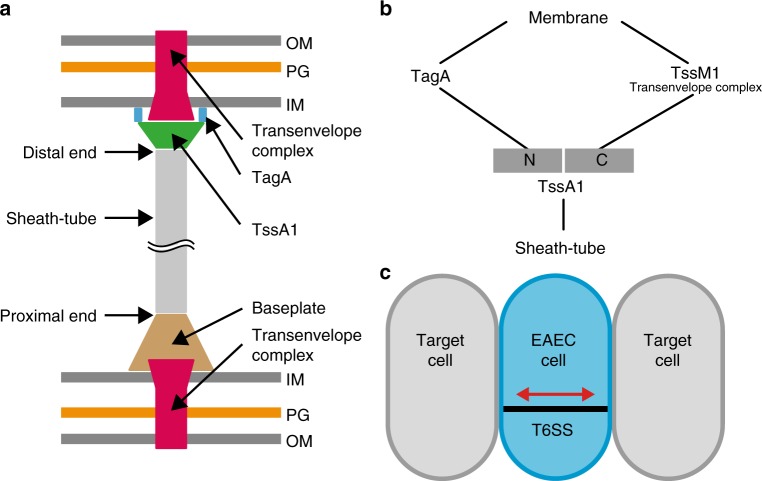
Interactions of both T6SS ends with the cell envelope mediate bidirectional contractions, which could be beneficial in a biofilm setting. **a** Shown is a schematic proposing a model for a cell-spanning EAEC T6SS-1. The sheath-tube proximal end is attached to the transenvelope complex via the baseplate. TssA1 mediates the termination of polymerization and the binding of the distal end to the cell envelope through interaction with TssM1 (transenvelope complex) and TagA (see also^22^). TssA1 along with TagA might act as an alternative baseplate that can trigger contraction at the distal end. **b** A diagram representing the interaction network at the T6SS distal end of a sheath-tube module. TssA1 is a central hub for anchoring the distal end to the membrane, which is essential for non-canonical contractions. TssA1 interacts with the transmembrane component TssM1 and membrane-associated TagA. Impairing either of the two links between TssA1 and the membrane (deletion of TssA C-terminus or TagA) results in sliding of the distal end of a growing T6SS structure. **c** The possibility to fire a given spanning T6SS in two opposite directions would be beneficial in densely populated biofilms to mediate interactions with neighbors on either cell side

In conclusion, we show that both ends of the EAEC T6SS-1 are bound to the cell envelope, which ultimately allows for bidirectional contractions. Our study also indicates the presence of an intricate mechanism for terminating sheath-tube polymerization, which is compatible with a recent report^22^. TssA1 is central for recruitment of the T6SS distal end to the cell membrane and requires interactions of TssA1 with both the transenvelope complex (TssM1) and TagA. Our model (Fig. 7a) is based on the following lines of evidence: (1) time-lapse imaging that revealed non-canonical contractions, (2) ECT imaging that revealed cell-spanning T6SSs and (3) the occurrence of bending only in spanning structures, indicating anchoring of both T6SS ends in the cell envelope. Furthermore, the C-terminal domain of TssA1 plays an important role in the processes at the distal end, since its absence led to (1) a significant decrease of non-canonical contractions, (2) the absence of the static extended phase, (3) longer T6SSs, and (4) a sliding of the polymerizing distal end against the inner membrane. The anchoring of the T6SS distal end in the cell envelope is mediated by different domains of TssA1 that interact with the transenvelope complex (TssJLM) and TagA (Fig. 7b). Interestingly, components of the TssJLM complex are dynamic (Supplementary Fig. 7 and ref. ^38^) and could facilitate the sampling of the inner membrane for approaching T6SS distal ends.

Our proposed model raises the important question of whether the contractile sheath could eject the Hcp tube during contraction toward the distal end. Extensive structural work on the T4 phage^39^, pyocins^40^, and T6SS^14,41,42^ have established a conserved contraction mechanism for CISs: structural rearrangements in the proximal end-bound baseplate trigger a conformational change in the proximal sheath subunits. This in turn initiates a conformational switch of the adjacent sheath subunits, propagating from the proximal end all the way toward the distal end. The movement of Hcp toward the proximal end is achieved by the increase of the sheath diameter, the loss of sheath-Hcp interactions, and the decrease of the sheath length at the proximal end. Similarly, we suggest that the triggering of sheath contraction at the distal end would result in the Hcp tube being pulled toward the distal end and could be ejected into extracellular space. We speculate that contraction at the distal end could be triggered by a complex consisting of TssA1, TagA and the transenvelope complex, which is consistent with the absence of non-canonical contractions in TssA1[1–392], TssA1[1–498], and ΔTagA mutants. Future studies will address the challenging task of tracing the fate of Hcp upon contraction toward the distal end.

Our study serves as a framework for understanding new concepts at the T6SS distal end. The data indicate in particular, that variations in the TssA C-terminal domain could manifest in mechanistic variations between T6SSs that are adapted to different functional roles and ecological settings. For example, we showed that the C-terminal extension EX of TssA1 is essential for non-canonical contractions in EAEC. EX, however, is absent from TssA in *V. cholerae*, being consistent with the absence of non-canonical contractions in this system. Like many other T6SSs, the EAEC T6SS-1 plays a role in biofilm formation^24,43,44^. The concept of a cell-spanning T6SS could essentially double the system’s attack range and allow for interactions with targets on either side of the cell (Fig. 7c), which would be especially beneficial in EAEC’s ecological niche—the crowded environment of the human intestine.

## Methods

### Plasmids, strains, and primers

Plasmids, strains and primers used in this study are listed in Supplementary Tables 1, 2 and 3, respectively. *E. coli* DH5α was used for all general cloning procedures. *E. coli* TOP10, *Vibrio cholerae* 2740–80 or enteroaggregative *Escherichia coli* (EAEC) 17–2 and its derivatives were used for all subsequent analyses.

### DNA manipulations

All DNA constructs were prepared using standard molecular biology techniques. Gene knockouts and fusions to fluorescent labels in EAEC 17–2 were carried out essentially as described before^45^.

### Light microscopy imaging

For imaging of Hcp1-CouAA, EAEC 17–2 strains were grown at 37 °C in M9 minimal media supplemented with 0.4% glycerol, 0.5 mM CouAA and relevant antibiotics to OD_600_ ~1.5. Cells were then induced with 0.2% arabinose (final concentration) and grown for 1.5 h for Hcp1-CouAA expression. Subsequently, cells were thoroughly washed with PBS and spotted on an agar pad. Images were taken on an FEI CorrSight microscope fitted with a Xe lamp, a 100 × 1.45 NA oil objective, a Hamamatsu Orca Flash camera (2k × 2k, 6.5 × 6.5 μm) and the following filter set: EX 377/50, BS 409, EM 466/40.

For imaging of chromosomal TssB1-sfGFP, cells of EAEC 17–2 *tssB1-sfgfp* and its derivatives were grown at 37 °C in M9 minimal media supplemented with 0.4% glycerol until OD_600_ ~1.5–2.0. Cells were then spotted on an agar pad. EAEC spheroplasts were prepared as previously described^17^. Images were taken using a Visitron Spinning Disk confocal microscope equipped with a 100 × 1.49 NA oil objective and a Hamamatsu Orca Flash camera (2k × 2k, 6.5 × 6.5 μm). Cell outlines (indicated as dashed lines) were determined by the signal of cytoplasmic sfGFP fluorescence.

For imaging of chromosomal sfGFP-TssM1, EAEC 17–2 *sfgfp-tssM1* cells were grown at 37 °C in M9 minimal media supplemented with 0.4% glycerol to OD_600_ ~1.5–2.0. Cells were then spotted on an agar pad and imaged using a Zeiss Spinning Disk confocal microscope using a 100 × 1.46 NA oil objective and an EM-CCD camera (512 × 512, 16 × 16 μm).

For imaging of *Vibrio cholerae* 2740–80, cells were grown as previously described^8^.

For the TssA1 membrane recruitment assay, *E. coli* TOP10 cells transformed with relevant plasmids were grown in LB to OD_600_ ~0.5 and induced with 0.003% arabinose and grown for further 45 min. Cells were then spotted on an agar pad. Images were taken using a Visitron Spinning Disk confocal microscope equipped with a 100 × 1.49 NA oil objective and an EMCCD iXon888 camera (1k × 1k, 13 × 13 μm).

For statistical analyses, normal distribution was confirmed and unpaired, two-sided *t*-test was used.

### Protein purification and mass spectrometry

Hcp1-wt-6xHis and Hcp1-CouAA-6xHis were expressed in EAEC 17–2 cells grown at 37 °C in LB or M9+CouAA medium, respectively. Cells were lysed in buffer A (50 mM Tris, 300 mM NaCl, 1 mM EDTA, 20 mM imidazole, pH 8.0) by sonication. The soluble fraction was loaded on a nickel column, washed thoroughly with buffer A and the protein was eluted by 0–1 M imidazole gradient. Peak fractions were pooled and subjected to dialysis against buffer B (20 mM Tris, 100 mM NaCl, pH 8.0) and stored at −80 °C.

Molecular weight of the purified proteins was determined by electrospray mass spectrometry (ESI-MS) at the Functional Genomics Center Zurich. Briefly, samples were desalted with ZipTip and the m/z data were deconvoluted into MS data using MaxEnt1 software with a resolution of the output mass 0.5 Da.

### Western blot analyses

Hcp1 expression and secretion were examined by Western blots using rabbit anti-Hcp1 polyclonal primary antibodies (GenScript) at a 1:10'000 dilution and goat anti-rabbit IgG conjugated with horseradish peroxidase (HRP) (Abcam, cat no: ab6721) at a 1:20'000 dilution and detected with ECL blotting reagent. To detect expression and secretion of His-tagged Hcp1, HRP-conjugated 6xHis Epitope monoclonal antibodies (Thermo Fisher, cat no: MA1–21315-HRP) were used at a 1:750 dilution.

### Hcp1-CouAA correlative light-electron microscopy at cryogenic temperatures

EAEC 17–2 Δ*hcp1* pEVOL pSZ232–8X strain cells were grown exactly as for room temperature light microscopy imaging purposes, mixed with protein-A-conjugated 10 nm gold beads (Cytodiagnostics) and applied to freshly glow-discharged 200 mesh Au Quantifoil (2/2) grids. The grids were plunge-frozen into a liquid ethane-propane mixture using an FEI Vitrobot (Mark IV) and stored in liquid nitrogen.

To identify relevant targets (cells harboring extended T6SS structures) for electron cryotomography, the grids were transferred to an FEI CorrSight microscope equipped with a cryostage and visualized using a 40 × 0.9 NA air objective and MAPS software.

### ECT imaging

For electron cryotomography, samples were imaged using an FEI Titan Krios TEM operating at 300 kV, equipped with a Gatan imaging filter set to zero-loss mode with a slit-width of ± 20 eV to remove inelastically scattered electrons. A Gatan K2 Summit direct electron detector was used for data acquisition with SerialEM software^46^. Cells were imaged at a magnification of 26’000, corresponding to a pixel size of 0.54 nm at the specimen level. Specimen were tilted from approximately −20° to +60° and −21° to −60° with 1° increments. The defocus was set to −10 µm (or 0 µm for imaging with Volta phase plate), and the total dose for each tilt series was ~150 e/Å^2^.

Tomographic reconstructions from tilt series were calculated using the IMOD software package^47^. Tilt series images were aligned using 10 nm gold beads as fiducial markers. Aligned stacks were produced with images binned four times resulting in the final pixel size of 2.16 nm. Tomograms were generated by back projection. All subsequent image analyses were carried out in IMOD.

## Supplementary information

Supplementary Information

Description of Additional Supplementary Files

Supplementary Movie 1

Supplementary Movie 2

Supplementary Movie 3

Supplementary Movie 4

Supplementary Movie 5

Supplementary Movie 6

Reporting Summary

Source Data

## Data Availability

Representative tomograms have been deposited at the Electron Microscopy Data Bank (EMDB) with accession codes EMD-4694, EMD-4695, EMD-4696 and EMD-4697. Source data for Fig. 5d and Supplementary Fig. 4a are provided as a Source Data file. Other data supporting the findings are available in the paper and its Supplementary Information files, or from the corresponding author upon request.
