# Supplementary material for: Bidirectional contraction of a type six secretion system
Source: Nat Commun. 2019 Apr 5;10:1565. doi: 10.1038/s41467-019-09603-1 (PMC6450956; doi:10.1038/s41467-019-09603-1)
Supplement: Supplementary file 1 — Supplementary Information [file 41467_2019_9603_MOESM1_ESM.pdf]

Supplementary Information

Bidirectional contraction of a type six secretion system

Piotr Szwedziak [1] and Martin Pilhofer [1,†]

[1] Institute of Molecular Biology & Biophysics, Eidgenössische Technische Hochschule Zürich, CH-8093 Zürich, Switzerland

† Corresponding author: Martin Pilhofer, Institute of Molecular Biology & Biophysics, Eidgenössische Technische Hochschule Zürich, CH-8093 Zürich, Switzerland, email: pilhofer@biol.ethz.ch, phone: +41 44 6333963

Supplementary Figures 1-7

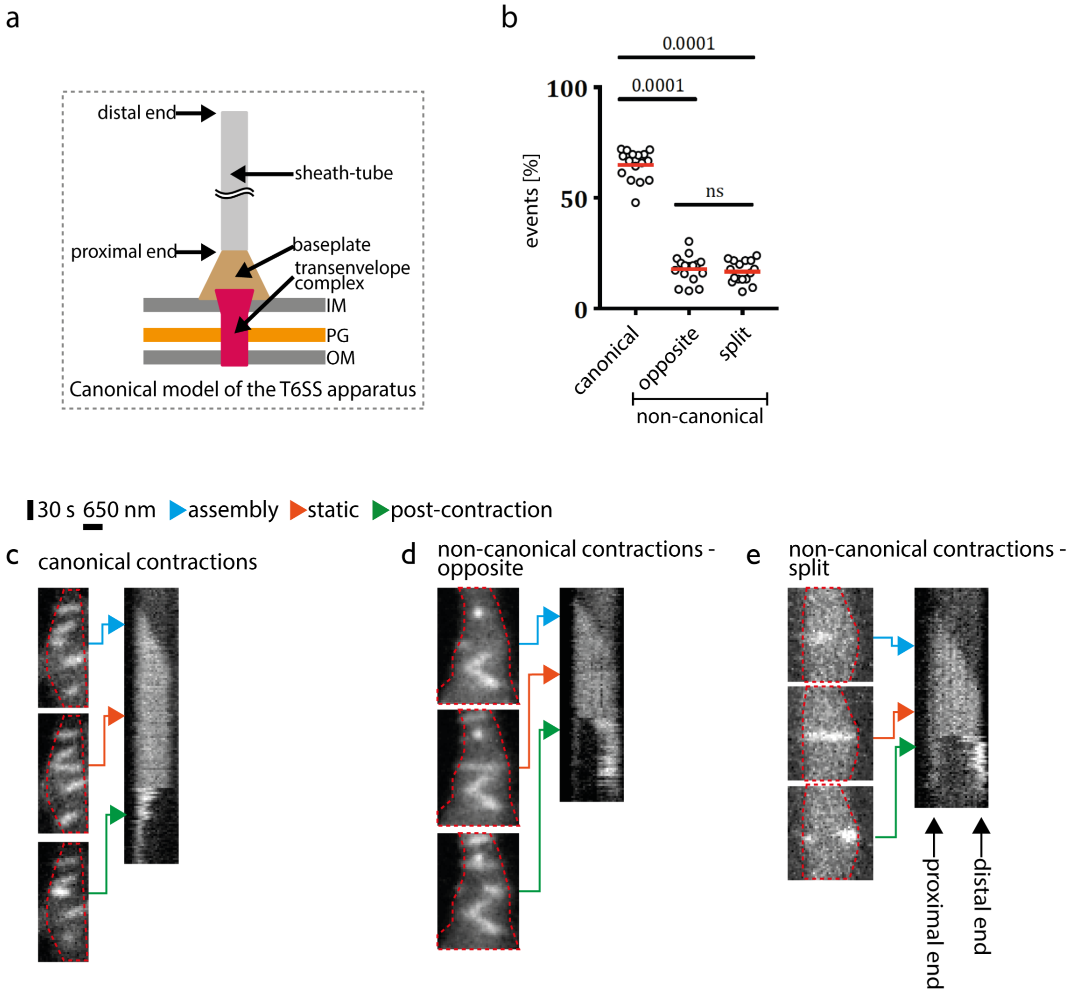

Supplementary Figure 1. EAEC TssB1-sfGFP dynamics.

(a) A schematic view of a T6SS apparatus with its proximal end anchored to the membrane-associated baseplate and distal end extending into the cytoplasm.

(b) Statistical analysis of TssB1-sfGFP dynamics in EAEC cells. Each data point represents one replicate of the imaging experiment. Red lines represent average values. Exemplary images are shown in Fig. 1.

(c-e) TssB1-sfGFP dynamics in EAEC spheroplasts also revealed the three classes of contractions (b, canonical; c, opposite; d, split). Spheroplasts were analyzed by time-lapse fluorescence imaging (1 frame every 3 s). For each example, three snapshots for

each cell are shown, representing assembly/static/post-contraction states, respectively. For each example, the three snapshots are correlated with a kymograph of a selected T6SS structure as indicated by arrows. Cell outlines are indicated by red dashed lines. Images were positioned to orient T6SS assembly start sites (proximal ends) on the left. See also Movie S3.

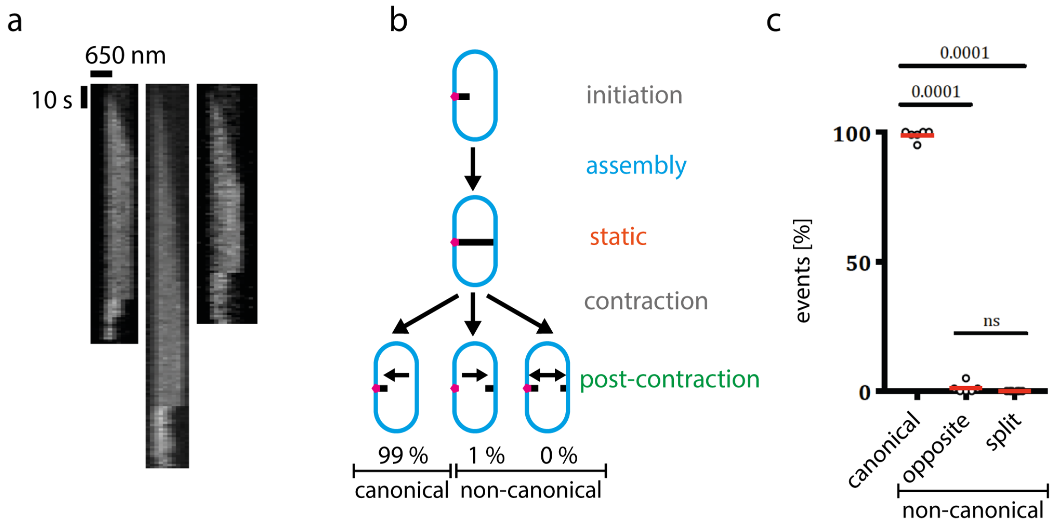

Supplementary Figure 2. TssB1-sfGFP dynamics in *V. cholerae* 2740-80.

TssB1(VipA)-sfGFP dynamics in *V. cholerae* 2740-80 cells only reveal canonical contraction events. Cells were analyzed by time-lapse fluorescence imaging (1 frame every 3 s). Shown are three representative kymographs (**a**), the classification/quantification of contractions (**b**; n=292), and the statistical analysis of the events shown in b (**c**). Each data point represents one replicate of the imaging experiment. Red lines represent average values. See also Movie S4.

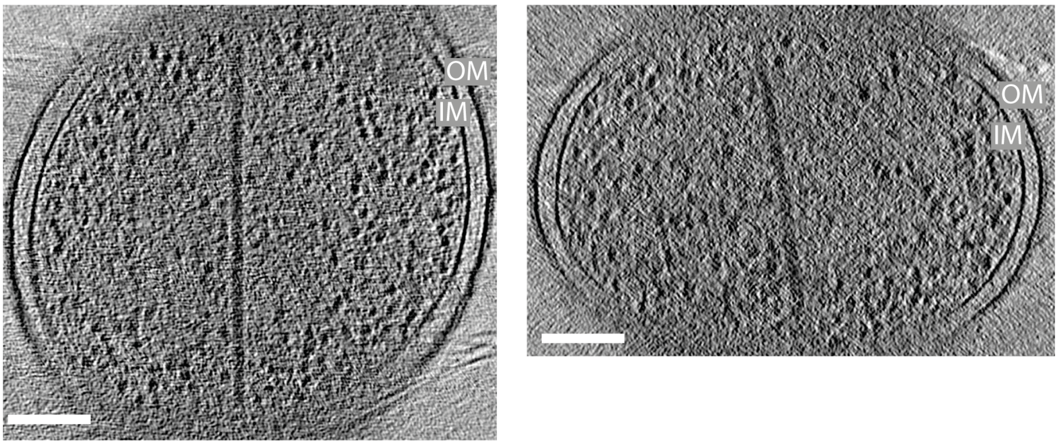

Supplementary Figure 3. Unclassified T6SSs imaged by ECT.

Shown are two examples of T6SSs in cryotomograms of EAEC in the TssB1-non-contractile background. The shown structures could neither be classified as spanning nor non-spanning, because their orientation in the direction of the electron beam resulted in the missing wedge of the data obscuring the ends of the structures. In Fig. 2c, such T6SSs were categorized as not classified.

Scale bar: 100 nm. Thickness of tomographic slices: 21.6 nm.

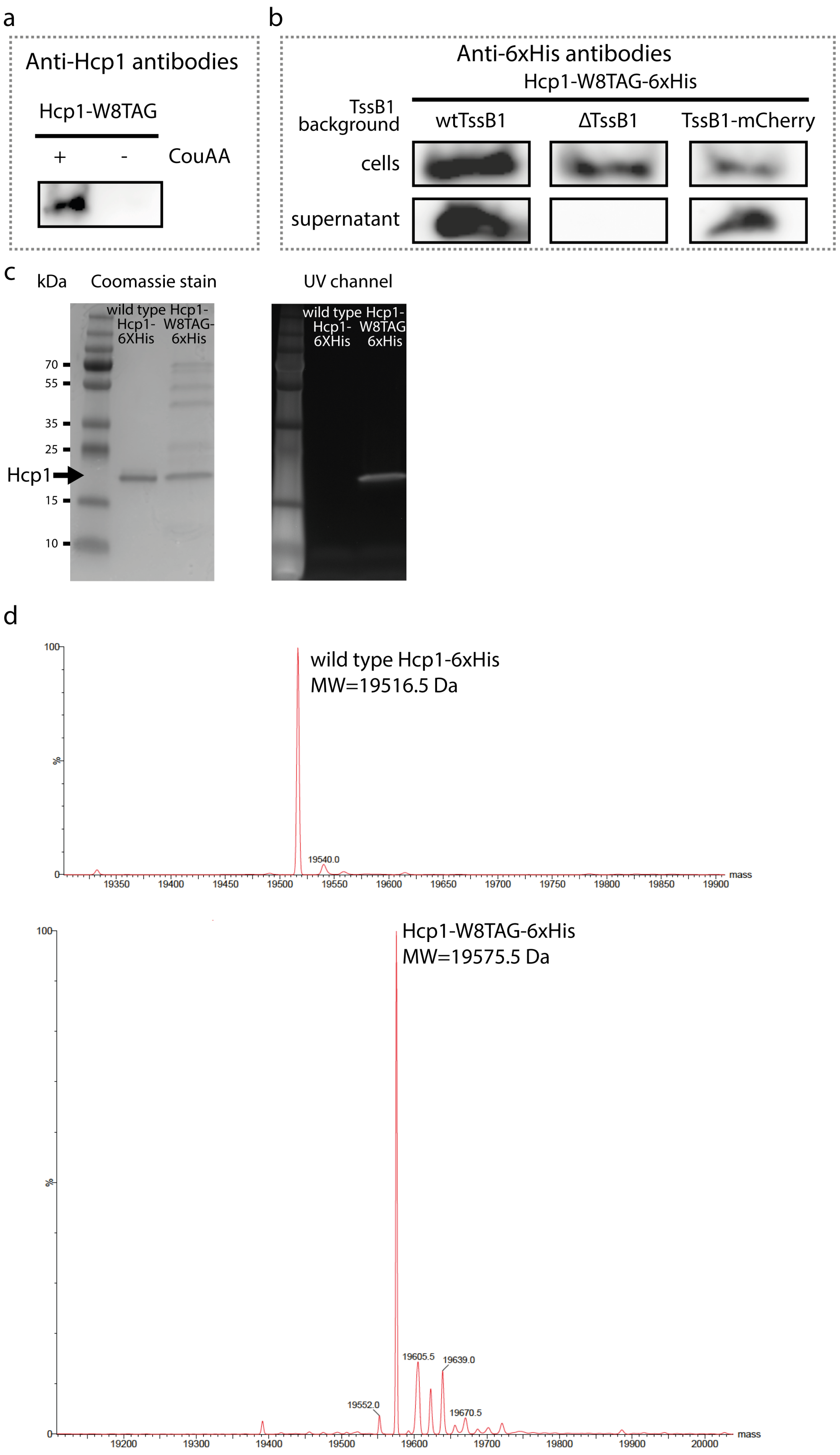
a
Anti-Hcp1 antibodies
Hcp1-W8TAG
+
-
CouAA
b
Anti-6xHis antibodies
Hcp1-W8TAG-6xHis
TssB1 background
wtTssB1
ΔTssB1
TssB1-mCherry
cells
supernatant
c
kDa
Coomassie stain
wild type Hcp1-6XHis
Hcp1-W8TAG-6xHis
70
55
35
25
Hcp1
15
10
UV channel
wild type Hcp1-6XHis
Hcp1-W8TAG 6xHis
d
wild type Hcp1-6xHis
MW=19516.5 Da
19540.0
Hcp1-W8TAG-6xHis
MW=19575.5 Da
19605.5
19639.0
19552.0
19670.5
%
mass
100
0

Supplementary Figure 4. Validation of Hcp1-labeling with CouAA by orthogonal translation.

(a) Western blot analyses showing that synthesis of Hcp1 with the amber codon in position 8 (Hcp1-W8TAG) was dependent on the presence of CouAA in the growth medium. For this experiment, Hcp1-W8TAG expression was probed in EAEC cells. Source data are provided as a Source Data file.

(b) Western blot analyses showing that Hcp1-CouAA-6xHis was secreted into the extracellular milieu (supernatant) in a TssB-dependent manner. All cells were grown in the presence of CouAA. For this experiment, C-terminally His-tagged Hcp1-W8TAG expression was probed in EAEC cells (top panels). For the analysis of supernatant, C-terminally His-tagged Hcp1-W8TAG was purified and then probed by western hybridization (bottom panels).

(c) Purified Hcp1-W8TAG-6xHis was fluorescent under UV light. Wild type Hcp1-6xHis and Hcp1-W8TAG-6xHis were purified from cultures (grown in the presence of CouAA), run on an SDS-PAGE gel, and imaged by Coomassie stain (to visualize the protein) and UV light (to visualize the fluorescence signal corresponding to CouAA). The CouAA fluorescence was specific to Hcp1-W8TAG-6xHis.

(d) Purified wild type Hcp1-6xHis and CouAA-labeled Hcp1-W8TAG-6xHis were subjected to electrospray ionization mass-spectrometry analyses, revealing that the orthogonal translation approach efficiently labeled all Hcp1. The theoretical molecular weight (MW) difference between wtHcp1-6xHis and CouAA-labeled Hcp1-W8TAG-6xHis is 59.02 Da (wtHcp1-6xHis MW=19517.24 Da, CouAA-labeled Hcp1-W8TAG-6xHis MW=19576.26 Da). The observed MW difference was 59 Da.

name	TssA1 construct	T6SS	sliding
wild type TssA1	ImpA_N, VasJ, EX; 1–532	+	-
TssA1[221-377]	221–377	-	n/a
TssA1[399-532]	399–532	-	n/a
TssA1[221-532]	221–532	-	n/a
TssA1[1-392]	1–392	+	+
TssA1[1-498]	1–498	+	+

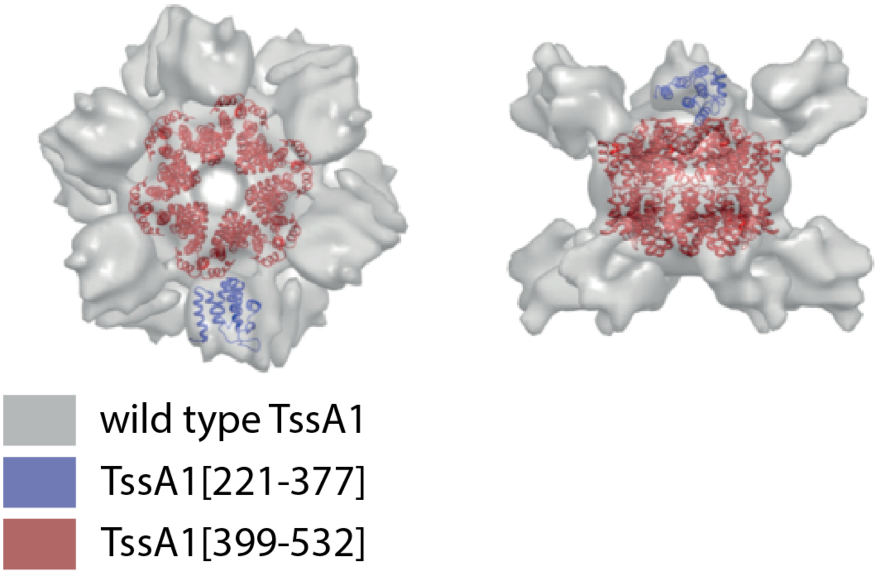

Supplementary Figure 5. Table summarizing the results of testing different TssA1 mutants for T6SS assembly.

The *tssA1* gene was deleted from the chromosome and different TssA1 constructs were supplied on inducible plasmids. T6SS assembly was monitored by imaging the fluorescence of GFP-tagged sheath ("+" in the T6SS column indicates the observation

of assembly and dynamics). TssA1[1-392] and TssA1[1-498] showed T6SS assembly and dynamics that were reminiscent of those observed in the *ΔtagA* background.

The bottom shows a negative stain EM reconstruction (~19 Å resolution) of wild type TssA1[1-532] (grey) with fitted crystal structures encompassing residues [221-377] (purple) and [399-532] (red) [adapted from [1]].

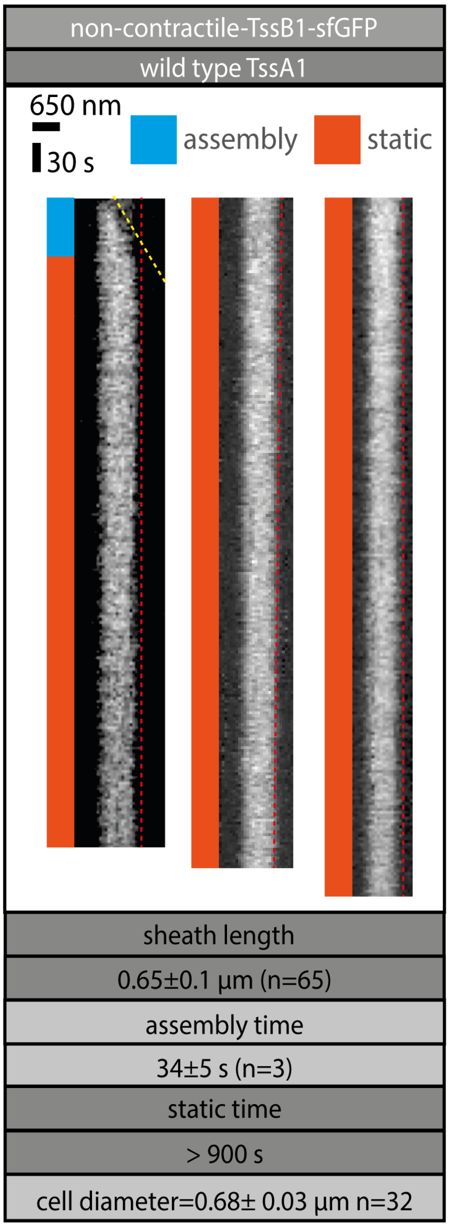

Supplementary Figure 6. Summary of the sheath dynamics in the wild type TssA1 and non-contractile TssB1-sfGFP background.

The visualized structures (shown are examples of kymographs) stayed indefinitely in the extended, static form, the average sheath length matched the wild type TssA1/TssB1-sfGFP background and no sliding against the IM was observed.

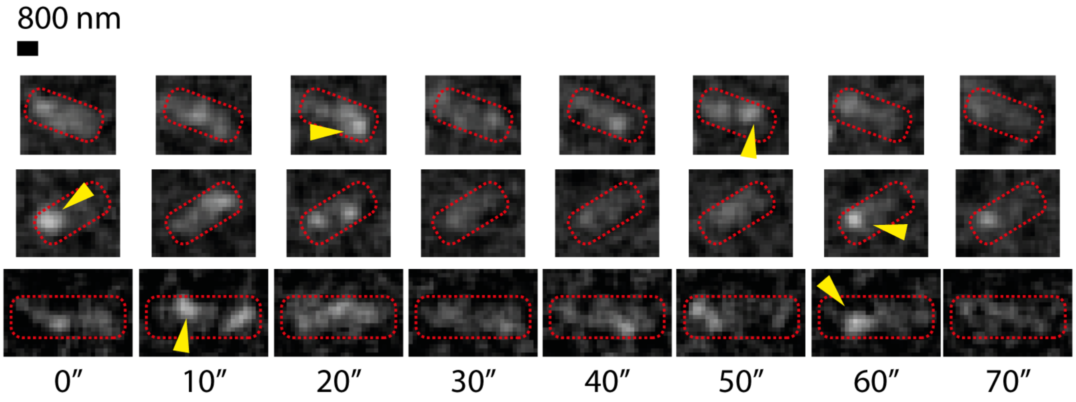

Supplementary Figure 7. sfGFP-TssM1 dynamics in EAEC cells.

Shown is time-lapse imaging of three cells (images recorded every 10 s). EAEC sfGFP-TssM1 localized into dynamic discrete foci (yellow arrowheads).

Supplementary Tables 1-3

Supplementary Table 1. List of plasmids used in this study

Name	Description	Resistance	Reference
pSZ232-wt	pBAD24::hcp1	Amp	This work
pSZ232-8X	pBAD24::hcp1(W8TAG)	Amp	This work
pSZ232-wt-His	pBAD24::hcp1-6xHis	Amp	This work
pSZ232-8X-His	pBAD24::hcp1(W8TAG)-6xHis	Amp	This work
pSZ245	pBAD24::tssA1[1-532]	Amp	This work
pSZ246	pBAD24::tssA1[221-377]	Amp	This work
pSZ247	pBAD24::tssA1[399-532]	Amp	This work
pSZ248	pBAD24::tssA1[221-532]	Amp	This work
pSZ249	pBAD24::tssA1[1-392]	Amp	This work
pSZ256	pBAD24::tssB1-non-contractile-sfGFP	Amp	This work
pEVOL	A plasmid encoding an aaRS/$tRNA_{CUA}$ pair specific for CouAA	Cam	[2,3]
pKD46	A plasmid encoding the λ Red recombinase	Amp	[4]
pKD4	A plasmid encoding an FRT-flanked Kan cassette	Amp, Kan	[4]
pSZ284	pBAD24::sfGFP-tssA1[1-532]	Amp	This work
pSZ285	pBAD24::sfGFP-tssA1[1-392]	Amp	This work
pSZ286	pBAD24::sfGFP-tssA1[1-498]	Amp	This work
pSZ272	pBAD24::tssA1[1-498]	Amp	This work
pSZ289	pBAD24::tssM1::sfGFP-tssA1[1-532]	Amp	This work
pSZ290	pBAD24::tssM1::sfGFP-tssA1[1-392]	Amp	This work
pSZ291	pBAD24::tssM1::sfGFP-tssA1[1-498]	Amp	This work
pSZ295	pBAD24::tssL1::sfGFP-tssA1[1-532]	Amp	This work
pSZ311	pBAD24::tagA::sfGFP-tssA1[1-532]	Amp	This work
pSZ312	pBAD24::tagA::sfGFP-tssA1[1-498]	Amp	This work
pSZ313	pBAD24::tagA::sfGFP-tssA1[1-392]	Amp	This work

Amp: ampicillin used at 100 μg/ml; Cam: chloramphenicol used at 50 μg/ml; Kan: kanamycin used at 25 μg/ml.

Supplementary Table 2. List of strains used in this study

Name	Description	Reference
E. coli DH5α	F-, Δ(*argF-lac*)U169, *phoA*, *supE44*, Δ(lacZ)M15, *relA*, *endA*, *thi*, *hsdR*	Thermo Fisher, cat no: 11319019
E. coli TOP10	F^{-} *mcr*A Δ(*mrr-hsd*RMS-*mcr*BC) φ80*lac*ZΔM15 Δ*lac*X74 *rec*A1 *ara*D139 Δ(*ara-leu*)7697 *gal*U *gal*K $λ^{-}$ *rps*L(Str^{R}) *end*A1 *nup*G	Thermo Fisher, cat no: C404050
Vibrio cholerae 2740-80 Δ*vipA* pBAD-VipA-sfGFP	*V. cholerae* 2740-80 deleted for the *vipA* gene and transformed with plasmid pBAD-VipA-sfGFP	5
17-2	Wild-type enteroaggregative *Escherichia coli* (EAEC)	6
17-2 Δ*hcp1* pEVOL pSZ232-wt	17-2 deleted for the *hcp1* gene and transformed with plasmids: pEVOL and pSZ232-wt	This work
17-2 Δ*hcp1* pEVOL pSZ232-8X	17-2 deleted for the *hcp1* gene and transformed with plasmids: pEVOL and pSZ232-8X	This work
17-2 Δ*hcp1 tssB1-mCherry* pEVOL pSZ232-8X	17-2 deleted for the *hcp1* gene and with mCherry inserted at the TssB1 C-terminus, transformed with plasmids: pEVOL and pSZ232-8X	This work
17-2 Δ*hcp1* Δ*tssB1* pEVOL pSZ232-8X	17-2 deleted for the *hcp1* and *tssB1* genes and transformed with plasmids: pEVOL and pSZ232-8X	This work
17-2 Δ*hcp1* Δ*tssC1* pEVOL pSZ232-8X	17-2 deleted for the *hcp1* and *tssC1* genes and transformed with plasmids: pEVOL and pSZ232-8X	This work
17-2 Δ*hcp1* pEVOL pSZ232-wt-His	17-2 deleted for the *hcp1* gene and transformed with plasmids: pEVOL and pSZ232-wt-His	This work
17-2 Δ*hcp1* pEVOL pSZ232-8X-His	17-2 deleted for the *hcp1* gene and transformed with plasmids: pEVOL and pSZ232-8X-His	This work
17-2 Δ*hcp1 tssB1-mCherry* pEVOL pSZ232-8X-His	17-2 deleted for the *hcp1* gene and with mCherry inserted at the TssB1 C-terminus, transformed with plasmids: pEVOL and pSZ232-8X-His	This work
17-2 Δ*hcp1* Δ*tssB1* pEVOL pSZ232-8X-His	17-2 deleted for the *hcp1* and *tssB1* genes and transformed with plasmids: pEVOL and pSZ232-8X-His	This work
17-2 Δ*tssB1*	17-2 deleted for the *tssB1* gene	7
17-2 Δ*tssC1*	17-2 deleted for the *tssC1* gene	This work
17-2 sf*gfp-tssM1*	sfGFP inserted at the TssM1 N-terminus	8
17-2 *tssB1-sfgfp*	sfGFP inserted at the TssB1 C-terminus	9
17-2 *tssB1-mCherry*	mCherry inserted at the TssB1 C-terminus	1

17-2 *tssB1-sfgfp ΔtssA1*	17-2 deleted for the *tssA1* gene and with sfGFP inserted at the TssB1 C-terminus	This work
17-2 *tssB1-sfgfp ΔtssA1* pSZ245	17-2 deleted for the *tssA1* gene and with sfGFP inserted at the TssB1 C-terminus and transformed with plasmid pSZ245	This work
17-2 *tssB1-sfgfp ΔtssA1* pSZ246	17-2 deleted for the *tssA1* gene and with sfGFP inserted at the TssB1 C-terminus and transformed with plasmid pSZ246	This work
17-2 *tssB1-sfgfp ΔtssA1* pSZ247	17-2 deleted for the *tssA1* gene and with sfGFP inserted at the TssB1 C-terminus and transformed with plasmid pSZ247	This work
17-2 *tssB1-sfgfp ΔtssA1* pSZ248	17-2 deleted for the *tssA1* gene and with sfGFP inserted at the TssB1 C-terminus and transformed with plasmid pSZ248	This work
17-2 *tssB1-sfgfp ΔtssA1* pSZ249	17-2 deleted for the *tssA1* gene and with sfGFP inserted at the TssB1 C-terminus and transformed with plasmid pSZ249	This work
17-2 Δ*tssB1* pSZ256	17-2 deleted for the *tssB1* gene and transformed with plasmid pSZ256	This work
17-2 *tssB1-sfgfp ΔtagA*	17-2 deleted for the *tagA* gene and with sfGFP inserted at the TssB1 C-terminus	This work

Supplementary Table 3. List of primers used in this study

Name	Sequence	Description
232iF	GAGGAATTCACCATGGCAATTCCAGTTTATCTGTG	to construct pSZ232-wt
232iR	AGCCAAGCTTGCATGTTACGCGGTGGTACGCTCAC	to construct pSZ232-wt
232vF	GTACCACCGCGTAACATGCAAGCTTGGCTGTTTTG	to construct pSZ232-wt
232vR	AACTGGAATTGCCATGGTGAATTCCTCCTGCTAGC	to construct pSZ232-wt
W8_F	CATGGCAATTCCAGTTTATCTGTAGCTGAAAGATGATGG CGGTGC	to construct pSZ232-8X
W8_R	GCACCGCCATCATCTTTCAGCTACAGATAAACTGGAATT GCCATG	to construct pSZ232-8X
Insert232_HisF	ATCATCATCATCATTAACATGCAAGCTTGGCTGTT	to construct pSZ232-His
insert232_HisR	GATCTTTTCTACGGGGTCTG	to construct pSZ232-His
Vector232_HisF	CAGACCCCGTAGAAAAGATC	to construct pSZ232-His
Vector232_HisR	TTAATGATGATGATGATGATGGGATCCCGCGGTGGTAC GCTCAC	to construct pSZ232-His
245vF	CGGGAAGTTCATGACATGCAAGCTTGGCTGTTTTG	to construct pSZ245
245vR	ATGAATGGAAGCCATGGTGAATTCCTCCTGCTAGC	to construct pSZ245 and pSZ249
245iF	AGGAGGAATTCACCATGGCTTCCATTCATTCGCTC	to construct pSZ245 and pSZ249
245iR	CCAAGCTTGCATGTCATGAACTTCCCGAATTGCAC	to construct pSZ245
246vF	AGCAGGTGATGTGACATGCAAGCTTGGCTGTTTTG	to construct pSZ246
246vR	GGTGATGGCAGACATGGTGAATTCCTCCTGCTAGC	to construct pSZ246 and pSZ248
246iF	GGAGGAATTCACCATGTCTGCCATCACCTCCGGAC	to construct pSZ246 and pSZ248
246iR	CAAGCTTGCATGTCACATCACCTGCTGCTGTATCC	to construct pSZ246

247vF	CGGGAAGTTCATGACATGCAAGCTTGGCTGTTTTG	to construct pSZ247 and pSZ248
247vR	AGATAAAATGTCCATGGTGAATTCCTCCTGCTAGC	to construct pSZ247
247iF	AGGAATTCACCATGGACATTTTATCTCTTGAGCCG	to construct pSZ247
247iR	CCAAGCTTGCATGTCATGAACTTCCCGAATTGCAC	to construct pSZ247 and pSZ248
249vF	TAACGATAACGTGACATGCAAGCTTGGCTGTTTTG	to construct pSZ249 and pSZ313
249iR	AGCTTGCATGTCACGTTATCGTTACAGAAGGTTCT	to construct pSZ249 and pSZ313
256vF	CAGACCCCGTAGAAAAGATC	to construct pSZ256
256vR	CGGTAACTCTACTTTCTTAAGCTCCACTTTCTTCTGCC	to construct pSZ256
256iF	CTTAAGAAAGTAGAGTTACCGCTCAAACTTCTGGTTGC	to construct pSZ256
256iR	GATCTTTTCTACGGGGTCTG	to construct pSZ256
284vF	GATGAGCTCTACAAAGCTTCCATTCATTCGCTCCT	to construct pSZ284, pSZ285, pSZ286
284vR	TTCACCTTTAGACATGGTGAATTCCTCCTGCTAGC	to construct pSZ284, pSZ285, pSZ286
284iF	GAATTCACCATGTCTAAAGGTGAAGAACTGTTCAC	to construct pSZ284, pSZ285, pSZ286
284iR	GAATGAATGGAAGCTTTGTAGAGCTCATCCATGCC	to construct pSZ284, pSZ285, pSZ286
272vF	CAAAAACTGAATGACATGCAAGCTTGGCTGTTTTG	to construct pSZ272 and pSZ312
272vR	ATGAATGGAAGCCATGGTGAATTCCTCCTGCTAGC	to construct pSZ272
272iF	AGGAGGAATTCACCATGGCTTCCATTCATTCGCTC	to construct pSZ272
272iR	AGCTTGCATGTCATTCAGTTTTTGCGGACTTCATA	to construct pSZ272 and pSZ312
289vF	CGGGAAGTTCATGACATGCAAGCTTGGCTGTTTTG	to construct pSZ289

289vR	TAGACATGGTGAATTCCTCCTCTGCAGTCAGTCAGTCTCCTCCACGG	to construct pSZ289, pSZ290, pSZ291
289iF	TGACTGCAGAGGAGGAATTCACCATGTCTAAAGGTGAAGAACTGTTCAC	to construct pSZ289, pSZ290, pSZ291
289iR	CCAAGCTTGCATGTCATGAACTTCCCGAATTGCAC	to construct pSZ289
290vF	TAACGATAACGTGACATGCAAGCTTGGCTGTTTTG	to construct pSZ290
290iR	AGCTTGCATGTCACGTTATCGTTACAGAAGGTTCT	to construct pSZ290
291vF	CAAAAACTGAATGACATGCAAGCTTGGCTGTTTTG	to construct pSZ291
291iR	AGCTTGCATGTCATTCAGTTTTTGCGGACTTCATA	to construct pSZ291
295vF	CGGGAAGTTCATGACATGCAAGCTTGGCTGTTTTG	to construct pSZ295
295vR	TAGACATGGTGAATTCCTCCTCTGCAGTTATCCCTGCCCGGTAAGCC	to construct pSZ295
295iF	TAACTGCAGAGGAGGAATTCACCATGTCTAAAGGTGAAGAACTGTTCAC	to construct pSZ295
295iR	CCAAGCTTGCATGTCATGAACTTCCCGAATTGCAC	to construct pSZ295
311vF	CGGGAAGTTCATGACATGCAAGCTTGGCTGTTTTG	to construct pSZ311
311vR	ACATGGTGAATTCCTCCTCTGCAGTCACTTCATGTCCCCTTGCG	to construct pSZ311, pSZ312, pSZ313
311iF	GTGACTGCAGAGGAGGAATTCACCATGTCTAAAGGTGAAGAACTGTTCAC	to construct pSZ311, pSZ312, pSZ313
311iR	CCAAGCTTGCATGTCATGAACTTCCCGAATTGCAC	to construct pSZ311
KanHcp1_F	ATGGCAATTCCAGTTTATCTGTGGCTGAAAGATGATGGCGGTGCAGATATCAAAGGTGTAGGCTGGAGCTGCTTC	To knock-out Hcp1
KanHcp1_R	TTACGCGGTGGTACGCTCACTCCATGCGTCGGAATGAATGATGTTGCCGTCCTTGTTATGAATATCCTCCTTAGTTCC	To knock-out Hcp1
KanTssC1_F	ATGCTGATGTCTGTACAGAAAGAAAAGAACGTTGCAGAGAGCGTGGTATCTGAAGGTGTAGGCTGGAGCTGCTTC	To knock-out TssC1
KanTssC1_R	GGATTGGCAAATCCTGCACCGCACCACCGGCCTGAGGCCCACGGATCTGAACACACTTATGAATATCCTCCTTAGTTCC	To knock-out TssC1

KanTssA1_F	ATTCATTCGCTCCTCAGTGCATGCCAGACGACACCCCGGGATGTGGCTGAACCGGCTCAGGGTGTAGGCTGGAGCTGCTTC	To knock-out TssA1
KanTssA1_R	GATGACTGGCTCCCCGGGCAAACATATCGTCACACTGTTCAAGCAGTGACAACCAGTTCTTATGAATATCCTCCTTAGTTCC	To knock-out TssA1
KanTagA_F	CGCGATGAAATCAGCAAACTGACTCACCCGGCACGCCCGGATGTGGACTGGCGTTACGTGGAGTGTAGGCTGGAGCTGCTTC	To knock-out TagA
KanTagA_R	ACCAGTGATGCCGGGTCAGCGCCACAATCAGAGCCAGCCCTTCATTCAGACCACTTAACTTATGAATATCCTCCTTAGTTCC	To knock-out TagA

Supplementary References

1. Zoued, A. et al. Priming and polymerization of a bacterial contractile tail structure. ***Nature*** 531, 59-63 (2016).

2. Charbon, G. et al. Subcellular protein localization by using a genetically encoded fluorescent amino acid. ***Chembiochem*** 12, 1818-1821 (2011).

3. Wang, J., Xie, J. & Schultz, P. G. A genetically encoded fluorescent amino acid. ***J Am Chem Soc*** 128, 8738-8739 (2006).

4. Datsenko, K. A. & Wanner, B. L. One-step inactivation of chromosomal genes in Escherichia coli K-12 using PCR products. ***Proc Natl Acad Sci U S A*** 97, 6640-6645 (2000).

5. Basler, M., Pilhofer, M., Henderson, G. P., Jensen, G. J. & Mekalanos, J. J. Type VI secretion requires a dynamic contractile phage tail-like structure. ***Nature*** 483, 182-186 (2012).

6. Aschtgen, M. S., Bernard, C. S., De Bentzmann, S., Lloubes, R. & Cascales, E. SciN is an outer membrane lipoprotein required for type VI secretion in enteroaggregative Escherichia coli. ***J Bacteriol*** 190, 7523-7531 (2008).

7. Brunet, Y. R., Henin, J., Celia, H. & Cascales, E. Type VI secretion and bacteriophage tail tubes share a common assembly pathway. ***EMBO Rep*** 15, 315-321 (2014).

8. Durand, E. et al. Biogenesis and structure of a type VI secretion membrane core complex. ***Nature*** 523, 555-560 (2015).

9. Logger, L., Aschtgen, M. S., Guérin, M., Cascales, E. & Durand, E. Molecular Dissection of the Interface between the Type VI Secretion TssM Cytoplasmic

Domain and the TssG Baseplate Component. ***J Mol Biol*** 428, 4424-4437 (2016).
