# Supplementary material for: Bidirectional contraction of a type six secretion system
Source: Nat Commun. 2019 Apr 5;10:1565. doi: 10.1038/s41467-019-09603-1 (PMC6450956; doi:10.1038/s41467-019-09603-1)
Supplement: Supplementary file 2 — Description of Additional Supplementary Files [file 41467_2019_9603_MOESM2_ESM.pdf]

Description of Additional Supplementary Files

File Name: Supplementary Movie 1
Description: A typical field of view showing TssB1-sfGFP dynamics in EAEC cells. Events classified as canonical contractions, non-canonical opposite contractions, and non-canonical split contractions are highlighted by the arrowheads.

File Name: Supplementary Movie 2
Description: Examples of three different kinds of TssB1-sfGFP dynamics observed in EAEC. Corresponds to the Fig. 1a-c.

File Name: Supplementary Movie 3
Description: Examples of the TssB1-sfGFP dynamics in EAEC spheroplasts. Corresponds to Supplementary Fig. 1c-e.

File Name: Supplementary Movie 4
Description: A typical field of view showing TssB1 (VipA)-sfGFP dynamics in V. cholerae 2740-80 cells. Three canonical events are highlighted by the arrowheads. Corresponds to Supplementary Fig. 2.

File Name: Supplementary Movie 5
Description: A typical field of view showing the TssB1-sfGFP non-contractile mutant dynamics in EAEC cells. Corresponds to Fig. 2.

File Name: Supplementary Movie 6
Description: Examples of T6SSs displaying the phenotype of the distal end sliding against the IM (blue arrowheads). TssB1-sfGFP dynamics were observed in the TssA1[1-392] background. Corresponding to cells that are depicted in Fig. 5a.
